# Development, scoring, and reliability for the Microscale Audit of Pedestrian Streetscapes for Safe Routes to School (MAPS-SRTS) instrument

**DOI:** 10.1186/s12889-024-18202-9

**Published:** 2024-03-06

**Authors:** Leigh Ann Ganzar, Katie Burford, Deborah Salvo, Chad Spoon, James F. Sallis, Deanna M. Hoelscher

**Affiliations:** 1 Michael and Susan Dell Center for Healthy Living, The University of Texas Health Science Center at Houston (UTHealth) School of Public Health Austin Campus, Austin, TX 78701 USA; 2 Department of Environmental Health Sciences, Mailman School of Public Health, Columbia University, 722 West 168th Street, New York, NY 10031 US; 3 Department of Kinesiology and Health Education, College of Education, The University of Texas in Austin, Austin, TX USA; 4 University of California San Diego, La Jolla, CA USA; 5 Herbert Wertheim School of Public Health and Human Longevity Science, University of California San Diego, La Jolla, CA USA; 6 Mary MacKillop Institute for Health Research, Australian Catholic University, Melbourne, Australia

**Keywords:** Environmental audit, Active commuting, Walkability, Physical activity, Children

## Abstract

**Background:**

Active commuting to school can be a meaningful contributor to overall physical activity in children. To inform better micro-level urban design near schools that can support active commuting to school, there is a need for measures that capture these elements. This paper describes the adaptation of an observational instrument for use in assessing micro-scale environments around urban elementary schools in the United States.

**Methods:**

The Micro-scale Audit of Pedestrian Streetscapes for Safe Routes to School (MAPS-SRTS) was developed from existing audit instruments not designed for school travel environments and modifications for the MAPS-SRTS instrument include the structure of the audit tool sections, the content, the observation route, and addition of new subscales. Subscales were analyzed for inter-rater reliability in a sample of 36 schools in Austin, TX. To assess reliability for each subscale, one-way random effects single-measure intraclass correlation coefficients (ICC) were used.

**Results:**

Compared to the 30 original subscales, the adapted MAPS-SRTS included 26 (86.6%) subscales with revised scoring algorithms. Most MAPS-SRTS subscales had acceptable inter-rater reliability, with an ICC of 0.97 for the revised audit tool.

**Conclusions:**

The MAPS-SRTS audit tool is a reliable instrument for measuring the school travel environment for research and evaluation purposes, such as assessing human-scale determinants of active commuting to school behavior and documenting built environment changes from infrastructure interventions.

**Supplementary Information:**

The online version contains supplementary material available at 10.1186/s12889-024-18202-9.

## Introduction

Physical activity provides numerous health benefits for children, including higher cardiorespiratory fitness, muscular fitness, bone health, and cardiometabolic health, as well as having a beneficial effect on mental health [1, 2]. To achieve these health benefits, it is recommended that children and adolescents aged 6 through 17 years should engage in at least 60 min of moderate-to-vigorous physical activity daily [3]. However, in the United States (U.S.), fewer than one quarter (24%) of children and adolescents meet these guidelines, leading to a need for strategies to increase daily physical activity among youth [4].

For all age groups, active travel has the potential to be a meaningful contributor to overall physical activity [5]. For children, promoting active commuting to school (ACS) can be an effective strategy to increase physical activity [6]. Investing in urban design features and transportation policies that support active travel and public transit use are 2 of the 8 intervention approaches that work for promoting physical activity [7, 8]. There was a time when ACS was common in the U.S., and thus, an important contributor to physical activity among children. However, car-centric urban design decisions and car-dependency have resulted in very low levels of participation in ACS, with only 10.9% of children age 5–17 walking or bicycling to school in 2017 compared to 47.7% in 1969 [9, 10]. This is even true for children who live within a reasonable active travel distance to school [10]. Additional reasons for the dramatic decline in ACS include that the distance from school that students live increased due to school preference and open transfer policies, urban sprawl, school siting guidelines, and small school closure [11–13]. Additionally, children’s independent mobility has decreased over the past decades, so fewer children are allowed to travel to/from school without adult supervision [14].

Parental perception of traffic safety is one of the primary reasons why even children living within walking or bicycling distance to school do not engage in ACS, and though these perceptions play a strong role on whether children actively commute to school or not, it is unlikely to improve perceptions if the reality of the environment is not safe and conducive to walking or biking [15]. So, measuring and improving built environments to become safe for ACS should always be the first step to actually optimize the real safety of the environment before trying to improve parental perceptions of the safety of the environment.

Many built environment elements are known to be associated with active travel among children, and most evidence is from macro-level environment features, including neighborhood residential density, road-network connectivity, and land-use mix, though there have been mixed findings about the associations between macro-level features and active transportation among children [15–17]. In addition to the macro-level features that encourage active travel, micro-scale factors are physical built environment design features along streets or segments and can provide qualities like comfort, safety, attractiveness that pedestrians and cyclists seek [18]. There are many physical components that make up the micro-scale built environment, including whether there are trees and shade along the route, the presence of safe crossings at intersections, stoplights that work, or whether the sidewalk is continuous and well-maintained [19, 20]. Micro-scale features are relevant for children traveling relatively small distances, such as to or from school, as they have the potential to influence perceptions of traffic safety, the major barrier to ACS as reported by parents. More specifically, lack of sidewalks, presence of sidewalk obstructions, and intersection features have been identified as top concerns for parents along routes their children could actively travel [21, 22] Safe Routes to School (SRTS) programs, often funded through an existing federal policy aimed at promoting safe active travel to school, often target micro-scale features near schools within engineering projects [23]. Despite the on-going and existing implementation of SRTS engineering projects near schools [24], there is little evidence to evaluate the specific microscale features that may help promote increases in ACS among children living within “active travel distance” to schools. This evidence would inform interventions like SRTS programs of modifiable urban design strategies and identify school environments that need to be prioritized.

There remains a need for a reliable and practical tool that can be used by researchers, practitioners, and community members to evaluate SRTS interventions, to document current conditions around schools to inform policy actions, to identify priority areas for investment, or to examine the moderating effect of the micro-level street environment on other types of interventions that promote ACS. There are several existing street audit tools to measure micro-scale characteristics specific to the school neighborhood environment or travel routes to school [25–28]. The Texas Childhood Obesity Prevention Policy Evaluation (T-COPPE) School Environment Audit Tool, a reliable instrument, captures the modifiable attributes of the built environments within school neighborhoods, but the authors did not determine a specific street sampling method for determining the school environment, which limits the utility of the tool [26]. Lee et al. (2020) adapted the T-COPPE audit tool and combined it with geographic information systems (GIS) measures of the macro-level built environment to develop a school walkability index within a 0.4 km Euclidean buffer [29]. However, the range of segments audited in this study was 15–87 segments within the buffer, indicating that audits for schools located in areas with high street connectivity would be resource intensive. Similarly, the Irvine-Minnesota Inventory (IMI) walkability audits assessed several micro-scale factors, including those related to traffic safety, accessibility, pleasurable settings, crime safety, density of housing, and diverse destinations [28]. However, this audit assessed individual children’s walking routes to school, making it resource intensive to gather enough information from enough home-school routes to make assessments about a school’s micro-scale environment. Lastly, Jones et al., (2010) developed a reliable and validated 44-item audit tool to assess aspects of the elementary school grounds but did not capture the street network around the schools [26]. As a result, this tool cannot be utilized within research seeking to understand how micro-scale environments impact active commuting to school behavior which takes place on streets near schools.

The Micro-scale Audit of Pedestrian Streetscapes (MAPS) direct observation tool was developed in 2013 to assess micro-level features of neighborhood walkability for general populations (i.e., without a specific focus on children). The MAPS tool has been previously validated as being related to active transport in all age groups [30, 31], but this version of the tool was developed for usage around individual’s homes on routes to various destinations, not the school environment. MAPS was the first audit tool to provide a systematic scoring system for summarizing data, and the intention of the tool was to be modified for different environments, countries, populations, and outcomes [31]. Pocock et al. (2020) adapted MAPS to develop the Microscale Audit of Pedestrian Streetscapes Global–School Neighbourhood (MAPS Global-SN) tool for adolescent school environments in New Zealand [27]. The MAPS Global-SN tool also requires GIS to determine the observation route, limiting the use of the tool those who have access to the software and required skills.

Overall, there remains a need for a school-specific, micro-scale observation instrument that is centered around the theme of ACS,provides reliable data, and could be used by researchers, practitioners, urban planners, schools, and community members to understand the micro-scale elements of the school neighborhood environment in the U.S. This school-specific tool would be particularly useful for evaluation of SRTS engineering projects, which are often focused on targeting micro-scale factors within school environments. Therefore, the objective of the present paper is to describe the adaptation of MAPS for use in assessing micro-scale school environments in an urban context and evaluate inter-observer reliability of the adapted tool, which can then be used to determine which micro-scale built environment features are associated with children’s ACS to support future planning and design standards for the school environment.

## Methods

### Setting and study design

This study used baseline data collected in 2018–2019 from elementary schools involved in the Safe Travel Environment Evaluation in Texas Schools (STREETS) study. The STREETS study is a five-year natural experiment that assesses the impact of Safe Routes to School infrastructure projects funded by a 2016 bond initiative from the City of Austin on children’s physical activity and ACS. The setting for this study was Austin, Texas, U.S.A, the capital city of the state, with a population of 907,779 in 2016, the year the bond initiative was passed, and which has been undergoing rapid urban expansion since the early 2000s [32, 33].

The STREETS study utilized a serial cross-sectional design to assess population-level changes in the prevalence of ACS in elementary schools in Central Texas. A subset of the schools recruited into the serial cross-sectional study were also recruited to be a part of a quasi-experimental, prospective cohort study to examine changes in child physical activity levels and psychosocial outcomes. This study used data from the 36 elementary schools recruited into the cohort study. Full methods of the STREETS study have been presented elsewhere [24].

### Micro-scale Audit of Pedestrian Streetscapes for Safe Routes to School (MAPS-SRTS) tool development

The Micro-scale Audit of Pedestrian Streetscapes for Safe Routes to School (MAPS-SRTS) tool was adapted from the MAPS direct observation (or audit) instrument [31] and the MAPS-Abbreviated instrument, a shorter version of MAPS tool [30]. The MAPS tool consisted of 120 micro-scale environmental items (e.g. presence and width of sidewalk, presence of sidewalk obstructions, shade coverage of sidewalks, and presence and quality of marked crosswalks) that potentially influence physical activity, and there were four sections used to measure different components of the streetscape: overall route, street segments, crossings, and cul-de-sacs. A street segment was defined as a section of street or road between two crossings on one side of the street (one block). A crossing occurred when the rater would travel through an intersection, whether a painted pedestrian crossing existed or not. Crossings were located between two segments and were coded any place two roads intersected.

In the MAPS tool, items collected for each section were summarized into subscales with either positive or negative valence scores. Audit data were collected by trained observers who followed a 0.25 mile route from each participant’s home address towards a pre-determined destination, which was typically a cluster of commercial locations or a park. The items and subscales from the MAPS tool had moderate to excellent inter-rater reliability (ICC values ≥ 0.41 and ≥ 0.60, respectively) [31]. MAPS-Abbreviated included 54 items from MAPS, which were selected by investigating the item-level partial correlations with physical activity across age groups (i.e., children, adolescents, younger adults, older adults), with a specific focus on walking or bicycling for transportation [30]. Items significantly correlated with active transport were included, but those with low frequency, limited policy relevance, or required too much labor to rate were dropped. Similar to the protocol for the full-length version of the MAPS tool, MAPS-Abbreviated collected audit data using 0.25-0.45-mile routes from participant homes towards predetermined destinations. The MAPS-Abbreviated and MAPS total scores have been reported to be strongly correlated with each other (*r* = 0.94) and with physical activity outcomes among children [30].

### Adaptations for MAPS-SRTS

MAPS-SRTS has several modifications from the MAPS and MAPS-Abbreviated tools for the purpose of assessment of the neighborhood environment immediately adjacent to and around schools, emphasizing features considered most relevant to the behavior of active traveling to/from school. Modifications were made to the (1) structure and content of the audit tool sections, (2) observation route, (3) and scoring.

#### Structure and content

Several adaptations to the MAPS-SRTS tool related to the structure and content were made to ensure suitability for the school environment. The adaptation of the MAPS tool was initiated by a SRTS practitioner (co-author CS) to ensure that important and appropriate school-specific built environment features were included, and the adaptation was coordinated closely with an investigator from the MAPS tool (co-author JS). First, a section was added to assess micro-scale characteristics of the street segment or segments where children enter and exit the building (i.e., the street segment adjacent to the school, usually directly in front of the school property). This new section, called the “school access segment,” had the same items and subscales as the original segments section. A school access segment was defined as the section of street directly in front of the main school entrance between two intersections. The school access segment was intended to cover the entire front of the school building (including any places children can access the entrance). For schools that had more than one school access segment, such as if there was an intersection across from the entrance of the school, or if there were multiple entrances to the school on different segments, then all school access segments were audited. Driveways on the school access segment that were school entrances or exits were counted as crossings but did not divide the school access segment into two. Because the destination of the route for MAPS-SRTS was always the school, the destination, land use, and cul-de-sacs sections were not included in the MAPS-SRTS scoring. These items were removed to reduce the length of the instrument tool, and though land use has been shown to be associated with ACS in children [34], this is not a readily modifiable component of the school neighborhood environment, nor is it within the scope of SRTS projects, and this tool was designed for use among researchers, planners, and practitioners implementing SRTS projects.

Because the environment around a school commonly has school-specific signage, several signage items were added to the school access segment section and segment section of the MAPS-SRTS tool to capture these elements. These items included: (1) the presence of school zone signage and (2) the presence of signage for a special speed zone during certain times of day (i.e. school drop-off and pick-up times).

#### Observation route

**Fig. 1:**
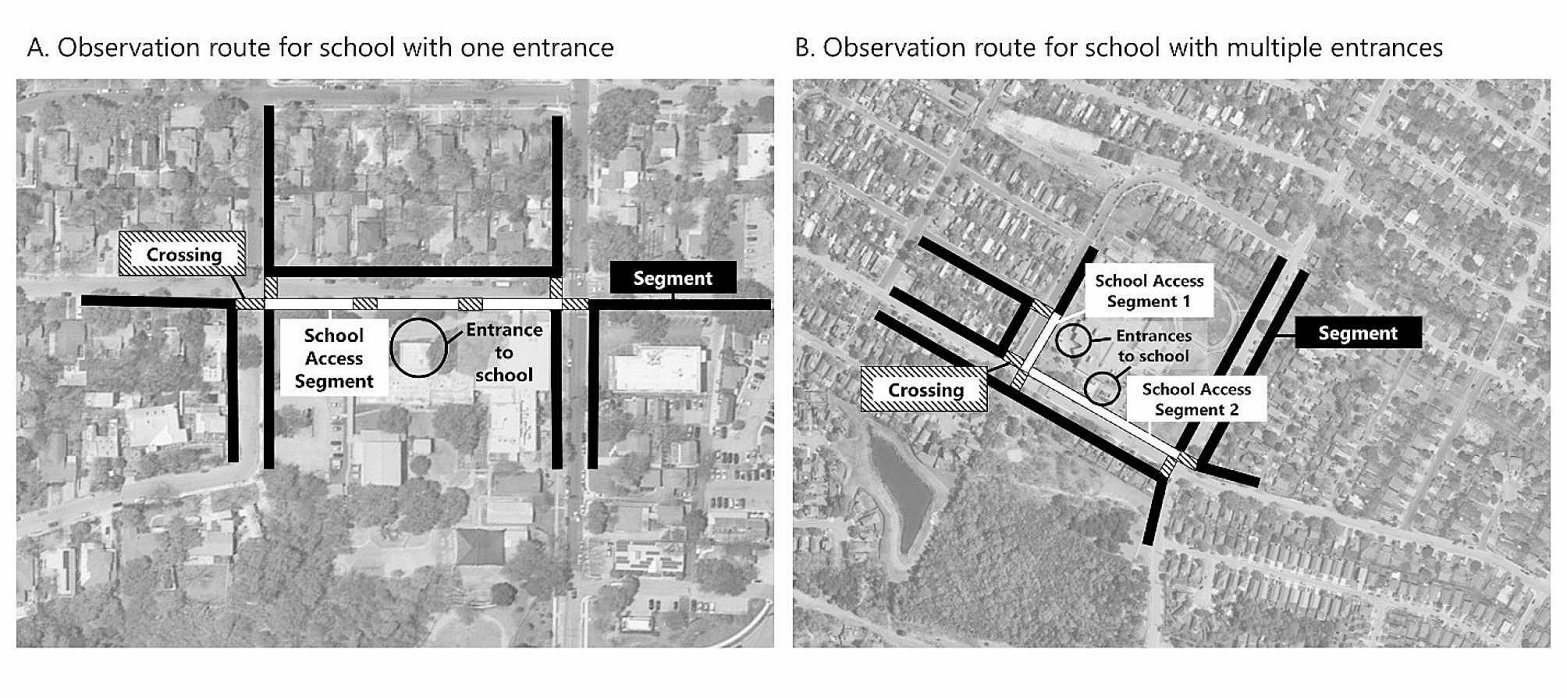
Example school observation routes for use with the Micro-Scale Audit of Pedestrian Streetscapes for Safe Routes to School (MAPS-SRTS)

For each school assessed using the MAPS-SRTS instrument, the observation route was first established (Fig. 1). The approach for route selection was based on ensuring measurement of segments and crossing that any kids that walk or cycle to school would have to use to access the school entrance. The observation route for each school began on the street where the school was located or the nearest street available, known as the school access segment, and the main school entrance was always the point of reference. The school entrance was confirmed by the study contact at each school. In addition to the school access segment, the route was determined using the “nearest-neighbor” method of spatial sampling [35]. This method used the following process for school observation route selection: (1) select all crossings that connect to the school access segment; (2) from each of those crossings, select all segments on one side of the street that connect to the crossing. Because it has been previously demonstrated that there is a high correlation between the features of both sides of the same street segment in micro-scale audit data, and to minimize data collection burden and make the tool practical for multiple audiences, including practitioners (non-researchers), it was decided to only measure one side of each street segment [27].This method captured the environment for those living within a very short distance (a few blocks) of school. While this sampling approach did not include all segments and crossings that a child would take to school, this route selection approach ensured that any route a child would take to school will include these components in any permutation or combination. This approach ensures that the final leg of the school journey was measured, and without an accessible final part of the trip, it does not matter how accessible the first parts of the route would be.

#### Scoring

The MAPS-SRTS tool consisted of 90 items divided into three sections for data collection and scoring: (1) school access segments (33 items), (2) other segments near schools (30 items), and (3) crossings (27 items). The school access segment examines the micro-scale characteristics of the road where children enter and exit the school building. The other segment section of the instrument characterizes the roads surrounding and leading to the school, and the crossing section assesses characteristics of where pedestrians cross the road or school driveway. These items were used to compute multiple subscales for each section.

**Fig. 2:**
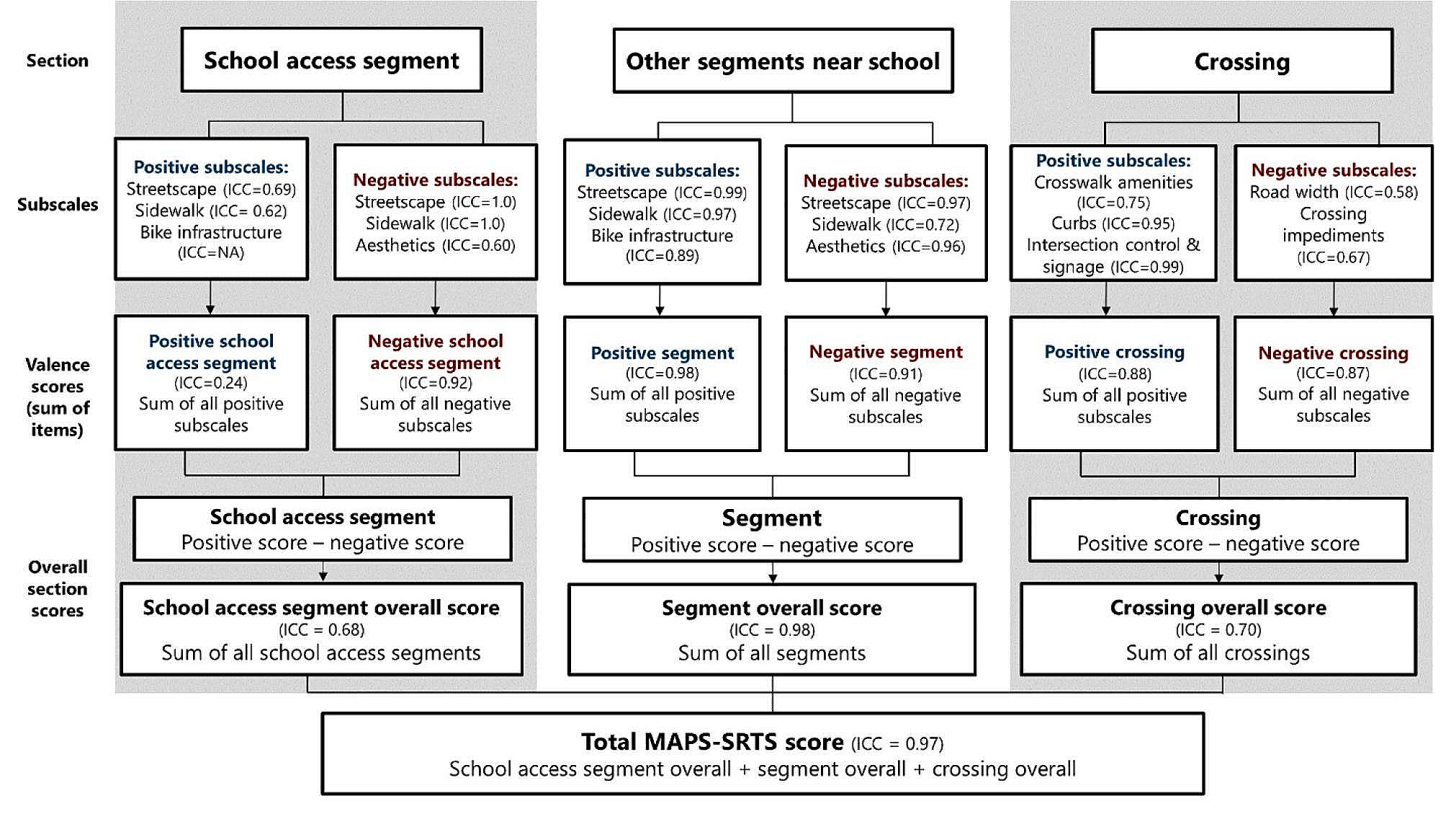
Scoring schema of subscales and total score for the Micro-scale Aaudit of Pedestrian Streetscapes for Safe Routes to School (MAPS-SRTS) instrument

The subscale development for the MAPS-SRTS instrument relies on a hierarchical scoring system, as in the MAPS tool, where items collected for each section (school access segments, other segments near schools, and crossings) were summarized into subscales at several levels of aggregation and were rated as positive or negative valence scores, as shown in Fig. 2. The positive and negative scores for each subscale are combined into an overall score for each section. The total MAPS-SRTS score is an aggregate score of the overall school access segments, other segments near schools, and crossings scores, where a higher score indicates a more supportive micro-scale built environment for walking and bicycling to school. The initial MAPS-SRTS scoring schema involved 30 subscales, in addition to the total MAPS-SRTS score, including 11 school access subscales, 11 other segments near school subscales, and 8 crossing subscales.

### Data collection procedures

#### Training

Prior to data collection, each data collector underwent training both in the classroom and in the field. During 2 h of classroom training, data collectors watched a pre-recorded video by one of the lead investigators on the STREETS project. The classroom training included the following: (1) definition of the School Neighborhood Environment, (2) how to create and use printed maps to guide school audits, (3) a review of the contents of MAPS-SRTS Training Manual and Picture Guide, (4) expectations in the field, (5) how to use Qualtrics (Qualtrics, Provo, UT) instruments on iPad mini tablets (Apple Inc., Cupertino, CA, USA) for data collection, and practice on the tablets. During 3 h of field training, each data collector underwent hands-on training for each route type and practiced using both the tablet and hard-copy map/protocol. Each data collector was trained in the field by two of the lead data collectors across two school audits and had to reach consensus with trainers prior to going out into the field independently.

#### Data collection

MAPS-SRTS baseline audits of 36 schools were completed between March 2019 and June 2021 by a total of 11 trained data collectors. During data collection, two raters audited each school. One person was responsible for entering data on the tablet, the other for managing the school neighborhood map and protocol. The two raters completed one or two school audits per day during the data collection period. Immediately following data collection, all data were uploaded to Qualtrics. The average time to complete audits was 77.8 min (SD = 29.5 min) per school, excluding travel time. To assess interrater reliability, 15% (*n* = 5) of schools were randomly selected and were independently assessed by two pairs of raters.

### Statistical analysis

Analysis for each subscale within the originally proposed MAPS-SRTS instrument (*n* = 31 subscales) included descriptive and reliability analyses. The mean and standard deviation of scores for each subscale were calculated. To assess inter-rater reliability for each subscale, one-way random effects single-measure intraclass correlation coefficients (ICC) were used for ordinal and continuous scales, and an ICC of 0.60 or higher was deemed acceptable reliability [36]. For a subscale originally proposed in the MAPS-SRTS instrument to be included in the final scoring, decision rules were implemented based on reliability and theoretical relevance. Subscale reliability scores were considered acceptable if ICC values were classified as moderate or higher (ICC = 0.60 or higher). For subscales that originally included items or subscales with low reliability (ICC below 0.60), we excluded those items or subscales from aggregate scoring. In cases where an item had low variability in one section (e.g. school access segment section) and the same subscale was reliable in another section (e.g. segment section), the item or subscale was retained if the items did not reduce the aggregate section score’s reliability below acceptable standards. Items removed due to low reliability were removed from subscales in a stepwise process to assess the effects on the overall reliability of the subscale.

Descriptive characteristics for each school were calculated using median and interquartile range. Geographic information systems (GIS) was used to construct measures of population density and street network connectivity within a 1-mile Euclidean buffer of each school address collected from 2020 U.S. Census Bureau five-year block group estimates and the Texas Department of Transportation Open Data Portal [32, 37]. Additionally, school characteristics from the 2018–2019 Texas Education Agency socio-demographics were used to describe the school sample, which included total enrollment and measures of the percentage of economically disadvantaged students (eligible for free or reduced lunch), racial and ethnic distribution as determined by the school district, percentage of students with limited English proficiency, and urbanicity [38].

## Results

The descriptive characteristics of the 36 schools in the sample are presented in Table 1. During the 2018–2019 school year, most of the students enrolled at the schools were Hispanic (median: 64%; IQR: 31%, 90% %), and most of the schools were classified as major urban schools (72%), which can be further explained by the population density (median: 647; IQR: 524, 694) and connectivity (median: 221; IQR: 169, 287) of streets within a 1-mile Euclidean buffer of each school.

**Table 1:** Descriptive characteristics of schools

Characteristic	*N* = 36
School level characteristics	
Total school enrollment (median (IQR))	478 (435, 610)
Percent students eligible for free/reduced lunch (median (IQR))	48 (26, 82)
Percent students with limited English proficiency (median (IQR))	27 [6, 53]
Percent student racial/ethnic distribution (median (IQR))	
African American	3 [2, 4]
Hispanic	64 (31, 90)
White, non-Hispanic	32 (6, 67)
Urbanicity (n (%))	
Major Urban	26 (72%)
Urban	10 (28%)
Rural	0 (0%)
Neighborhood level characteristics	
Population density (per sq.km,(median (IQR)))	647 (524, 694)
Connectivity (number 3- & 4- way intersections,(median (IQR)))	221 (169, 287)
Number of school access segments audited (median (IQR))	1 [1, 2]
Number of other segments near school audited (median (IQR))	9 (7.75, 10)
Number of crossings audited (median (IQR))	7 [6, 8]

### Reliability results

There were 30 subscales and the total MAPS-SRTS score that were tested for reliability in the initial MAPS-SRTS scoring framework using data from the 5 randomly selected schools from the sample. In initial reliability analyses, 22 out of 30 subscales had acceptable reliability, including five school access segment subscales, nine other segments near school subscales, seven crossing subscales, and the total MAPS-SRTS score. Eight out of the 30 subscales tested had ICC results that fell below the threshold of acceptable reliability in initial analyses, or the subscale lacked sufficient variability for reliability analyses. Five subscales from the school access segment section had reliability estimates that were below the threshold or lacked variability as denoted by an ICC of “n/a”: the positive buffer subscale (ICC = 0.27, 95% CI = -0.62, 0.89), the positive shade subscale (ICC = n/a), the positive bicycle infrastructure subscale (ICC = n/a), the overall positive school access segment subscale (ICC = 0.19, 95% CI = -0.67, 0.86), and the overall school access segment subscale (ICC = 0.57, 95% CI = -0.33, 0.94). Two subscales in the other segments near schools section were below acceptable reliability: the positive buffer subscale (ICC = -0.22, 95% CI = -0.84, 0.71) and the positive shade subscale (ICC = -0.13, 95% CI = -0.81, 0.76). The crossing section had one subscale with an initial reliability estimate below the acceptable threshold, which was the road width subscale (ICC = 0.25, 95% CI = -0.63, 0.88).

Once initial reliability analyses were complete, inclusion of subscales in the final scoring was determined. Based on low reliability, there were four subscales removed from the MAPS-SRTS scoring schema: the positive buffer and positive shade subscales in the school access segment section and the positive buffer and positive shade subscales in the other segments near schools section. Once these subscales were removed, the overall school access segment and overall crossing subscales had acceptable reliability. The positive bicycle infrastructure subscale in the school access segment lacked sufficient variability for a reliability estimate. But the positive bicycle infrastructure subscale in the segment section, which includes the same items and scoring, was a reliable subscale (ICC = 0.89, 95% CI = 0.38, 0.99), and therefore this subscale was kept in the school access segment section. Additionally, the overall positive school access segment subscale had low reliability due to the lack of variability, but because the subscale consisting of the same items in the segment subscales showed acceptable reliability (ICC = 0.98, 95% CI = 0.90, 0.99), the subscale was retained in the school access segment section. There was one subscale (road width) that was revised due to initial low reliability results (ICC = 0.25, 95% CI = -0.63, 0.88). The initial road width subscale in the crossing section used two items (number of travel lanes and number of turn lanes) and trichotomized the sum of the two items. We recoded the subscale to be a dichotomous variable (≤ 4 lanes and > 4 total lanes) based on evidence that roads with more than 4 lanes increase the likelihood of pedestrian crashes [39]. With this revision, the subscale had higher reliability and was included in the negative crossing subscale (ICC = 0.58, 95% CI = -0.33, 0.95).

After removal of four subscales, there were 26 final subscales that were included in the MAPS-SRTS instrument, in addition to the total MAPS-SRTS score, as shown in Table 2, which includes the final revised reliability measures for each subscale and the total score. These subscales were included in the final scoring schema, as shown in Fig. 2.

**Table 2:** Micro-scale audit of pedestrian streetscapes– safe routes to school (MAPS-SRTS) subscale characteristics and final reliabilities

Subscale	Mean score (SD) *N* = 36 schools	# items or subscales	Sample items and overall subscale descriptions	ICC (95% CI) *N* = 5 schools
**School access segment subscales**				
Positive streetscape	6.50 (2.89)	11	Presence of public transit, posted speed limits, speed restrictions traffic calming, instructional signs, streetlights, street amenities	0.69 (-0.15, 0.96)
Positive sidewalk	7.22 (3.85)	3	Sidewalk presence and width	0.62 (-0.27, 0.95)
Positive bicycle infrastructure	0.11 (0.32)	1	Marked bicycle lane and physical barrier	N/A
Overall positive school access segment	13.83 (6.41)	3 subscales	Positive streetscape, positive sidewalk, and positive bicycle infrastructure subscales	0.24 (-0.64, 0.88)
Negative streetscape	0.97 (0.88)	2	High speed limits, presence of drivers	1.0 (N/A)
Negative sidewalk	0.31 (0.52)	2	Non-continuous sidewalk, major trip hazards	1.0 (N/A)
Negative aesthetics	0.11 (0.32)	1	Signs of neglect, such as graffiti or poorly maintained buildings	0.60 (-0.28, 0.95)
Overall negative school access segment	1.39 (1.10)	2 subscales	Negative streetscape, negative sidewalk, and negative aesthetics subscales	0.92 (0.54, 0.99)
Overall school access segment	12.44 (6.20)	2 subscales	Overall positive– Overall negative subscales	0.68 (-0.18, 0.96)
**Other segments near school**				
Positive streetscape	33.06 (9.18)	11	Presence of public transit, posted speed limits, speed restrictions traffic calming, instructional signs, streetlights, street amenities	0.99 (0.97, 1)
Positive sidewalk	35.50 (12.01)	3	Sidewalk presence and width	0.97 (0.80, 0.99)
Positive bicycle infrastructure	1.14 (1.78)	1	Marked bicycle lane and physical barrier	0.89 (0.38, 0.99)
Overall positive segment	69.69 (19.24)	4 subscales	Positive streetscape, positive sidewalk, and positive bicycle infrastructure subscales	0.98 (0.90, 0.99)
Negative streetscape	9.50 (2.71)	2	High speed limits, presence of drivers	0.97 (0.82, 0.99)
Negative sidewalk	3.17 (2.52)	2	Non-continuous sidewalk, major trip hazards	0.72 (-0.09, 0.97)
Negative aesthetics	1.56 (2.22)	1	Signs of neglect, such as graffiti or poorly maintained buildings	0.96 (0.83, 0.99)
Overall negative segment	12.67 (3.37)	3 subscales	Negative streetscape, negative sidewalk, and negative aesthetics subscales	0.91 (0.49, 0.99)
Overall segment	57.03 (18.65)	2 subscales	Overall positive– Overall negative subscales	0.98 (0.87, 0.99)
**Crossings**				
Positive crosswalk amenities	21.25 (6.03)	7	Crossing aids, marked crosswalk, high visibility striping, stop lines or crosswalk warnings, raised crosswalk, different material than road, protected refuge islands, curb extensions, bicycle box	0.75 (-0.03, 0.97)
Curbs	10.97 (3.92)	2	Ramp lines up with crossing	0.95 (0.67, 0.99)
Positive intersection control and signage	5.06 (3.52)	8	Yield signs, stop signs, traffic signal, traffic circle, green arrows for turn lane, pedestrian walk signals, push buttons, countdown signal	0.99 (0.90, 0.99)
Overall positive crossing	36.94 (10.21)	3 subscales	Positive crosswalk amenities, curbs, and positive intersection control and signage subscales	0.88 (0.37, 0.99)
Road width	0.31 (0.47)	2	Number of travel and turn lanes	0.58 (-0.33, 0.95)
Negative crossing impediments	9.17 (4.53)	4	No ramp pre- and post-crossing curb, crossing in poor condition	0.67 (-0.18, 0.96)
Overall negative crossing	9.28 (4.78)	2 subscales	Road width and negative crossing impediments subscales	0.87 (0.30, 0.98)
Overall crossing	27.44 (10.13)	2 subscales	Overall positive– Overall negative subscales	0.70 (0.13, 0.96)
**Total MAPS-SRTS score**	96.92 (26.22)	3 subscales	Sum of overall school access segment, overall segment, and overall crossing	0.97 (0.81, 0.99)

## Discussion

This study developed the MAPS-SRTS audit tool for the assessment of micro-scale street-level features in school neighborhood environments and determined the reliability of the MAPS-SRTS tool within elementary school neighborhoods in central Texas. After revisions, results showed that out of the 30 original subscales, 26 subscales (86.7%) were retained in the final MAPS-SRTS tool. Once the four subscales with low reliability were removed from the MAPS-SRTS tool, the total MAPS-SRTS score had excellent reliability (ICC = 0.97). As a result, we suggest that the final version of the MAPS-SRTS tool that only retains subscales with high reliability and is presented within this study be utilized by researchers and practitioners aiming to assess how supportive the micro-scale environment surrounding schools is for active travel.

The total reliability score of the MAPS-SRTS tool was comparable to two existing audit tools developed for school environments, which both had moderate to high inter-rater reliability [25, 27]. The ICC for the TCOPPE School Environmental Audit Tool and MAPS Global-SN were 0.602 and 0.97, respectively. While the TCOPPE Audit tool was developed within elementary school environments in Texas similar to the MAPS-SRTS tool, the items and scoring of the tool were not comparable to those used within the MAPS-SRTS tool [25]. In contrast, the MAPS Global-SN and the MAPS-SRTS were developed from the MAPS direct observation instruments [31, 40]. MAPS-SRTS and MAPS Global-SN both had high reliability scores for the positive streetscape subscale, the overall segment score, and the overall crossing score. However, the MAPS Global-SN was developed within secondary schools in New Zealand, and thus the modifications of the tool were made based on this context (e.g., round flashing orange lights, transportation facilities for adolescents) [27]. As the MAPS-SRTS tool was developed within an elementary school context in the U.S., both tools need to be adapted within more diverse geographic contexts.

Out of the 30 subscales within the MAPS-SRTS tool, we removed four subscales based on low reliability scores. For the school access segment and other segments near schools, the positive buffer subscale and shade subscale were removed completely for each section. The road width item was also revised because of low initial reliability, which was due to data collectors interpreting travel lanes versus turn lanes differently. The lower inter-rater reliability results for these subscales led to a recommendation that improved training protocols may be warranted to improve the reliability of these subscales. This training could include showing more visual image examples during the classroom training, practicing and quizzes with virtual street view examples, and ensuring high accuracy between test audit results of individual data collectors and lead data collectors during field training.

There may be other opportunities to improve upon the measurement of the school neighborhood environment. Based on the findings, we have several recommendations for iterations of the MAPS-SRTS tool and within future assessment efforts. First, to improve the reliability of the MAPS-SRTS tool we present several considerations for the buffer and shade items. In the original MAPS-SRTS tool, a buffer was determined to be present if there was a sidewalk separated from a roadway by a parking lane, and this item was included within the positive subscale. Within the planning literature, there is an ongoing debate about the benefits or risks of on-street parking on the pedestrian environment, which depends on street-design and context [41, 42]. For example, there is evidence to suggest that on major streets, on-street parking is dangerous for pedestrian safety but on minor streets, on-street parking can slow traffic speeds and traffic volume [41, 43]. Transportation researchers have recommended the prohibition of on-street parking near schools, and thus a buffer created from on-street parking may need to be included as a negative subscale rather than a positive subscale in this context [43].

Another construct for which measurement needs to be improved at the micro-scale is shade. The shade subscale, measured within the tool as the percentage of the length of the sidewalk/walkway covered by tree canopy or awnings, was also removed from this study due to a low reliability score. Auditors were asked to report a percentage of the sidewalk covered by shade (1–25%; 26–75%; 76–100%), which may have varied based on the time of day or season these data were collected. Future studies could improve the reliability of this measure by ensuring inter-rater assessments were completed during the same time period, and perhaps during times in which children actively commute to and from school. Audit-based measures of shade could be augmented with air temperature/relative humidity sensors, which can be used to develop thermal profiles to measure the environment children are walking and bicycling through [44]. As climate change is causing an overall rise in temperature and the intensity, duration, and frequency of heat waves [45], developing valid and reliable measures of environments that support safe physical activity of children in high temperatures is a pressing area for future research.

A further recommendation when measuring the micro-scale characteristics of a school neighborhood is to consider the power dynamics and inclusion of lived experience when deciding whether to include negative neighborhood aesthetics as a measure in the audit tool. The MAPS-SRTS and other audit tools include an item that measures perceptions of signs of neglect or physical disorder in the neighborhood, such as graffiti, poorly maintained buildings, or abandoned buildings, that is used for a negative aesthetics subscale. While there is evidence of associations between neighborhood disorder and lower physical activity in children [46], it is also worth noting that associations of physical disorder with health outcomes may be misleading because they lack inclusion of relevant covariates such as socioeconomic status and collective efficacy [47]. According to the necessity- versus choice-based physical activity models framework, children from low and middle income countries or deprived settings may be operating out of necessity based active commuting because they have no other option, even in unsafe travel conditions [48]. Additionally, the use of this item has the potential for significant researcher prejudice and bias, as typically the academic-based researchers conducting audits are highly educated, which is often used as a proxy for socioeconomic status, so especially for studies in low-income areas, the researchers may have different lived experiences and norms/values than those living in the neighborhoods being audited. So, while the negative aesthetics subscale is a reliable measure in MAPS-SRTS, we recommend the removal of the negative aesthetics when the MAPS-SRTS tool is being conducted by people who are not a part of the community they are auditing, as community members’ familiarity with the community could cause them to be more or less sensitive to physical disorder or aesthetic features than researchers who do not reside in the community [49]. If this tool is being used in a community-engaged and driven research project, the negative aesthetics subscale can be included in consideration of the representation, inclusion, and lived experiences of community members and residents engaged in the efforts to collect the data, a key principle in community engagement [50].

The MAPS-SRTS tool was developed to consider objective measures of the environment, which does not explain the experience, attitudes, and behaviors of those using and living in the environment. Future studies should seek to incorporate children’s and parents’ perspectives on the specific characteristics of the school travel environment, which could be informative for developing interventions and promoting active travel. Participatory mapping and qualitative GIS methods are two examples of participatory methods that could be used to engage children and parents [51, 52]. Wilson and colleagues (2019) used these methods to understand environmental barriers and facilitators of children’s active school travel, which included safety, material, and affective features [52].

In addition to improvements in the MAPS-SRTS tool, there were several limitations specific to the design and methods of this study. The first was inclusion of a small sample (*N* = 36) of urban and suburban elementary school neighborhoods within one region of the U.S. Due to the low sample size of schools, there were few schools (*n* = 5) included within the reliability analysis, which may have resulted in lack of variability of some items for the school access segment. Future research should assess the MAPS-SRTS tools’ psychometric properties across a greater range of school districts, including suburban and rural geographic regions. Additionally, one promising avenue for future research could be to consider more time-efficient audit-based methods, such as virtually through Google Street View, which have been validated multiple times [53–55]. This could also allow for improving the external validity of the existing tool by allowing researchers to capture more diverse geographic contexts than one metropolitan area. For example, the application of the MAPS-SRTS tool to measure the micro-scale environment across rural schools could add to the limited evidence that has identified strategies to promote ACS in rural areas [56]. While data collection for the present study did not take place during peak periods of travel (i.e., drop off and pick-up from school), the items included within MAPS-SRTS consider the existing built environment, which would not be influenced by travel behaviors, though the supports for active travel can change during drop off and pick-up from school (e.g. crossing guards), so future research should consider differences in what is available in the built environment versus how and whether those supports are used. Lastly, we did not examine the validity of the MAPS-SRTS tool with active commuting to school or physical activity outcomes, which is an important next step for researchers in evaluation efforts of SRTS engineering projects. we are not assessing validity with this manuscript, and that is beyond scope of this paper. The MAPS instrument from which all items, scales, and sections were drawn has been extensively validated with this type of analysis in many population groups, including children [30, 57].

The MAPS-SRTS was developed with practitioner usage in mind for implementing and evaluating Safe Routes to School interventions. Previous MAPS audits are not suitable for school-specific measurement of the streetscape or require specialized GIS skills to determine a buffer to audit [27, 31]. The MAPS-SRTS audit tool does not require specialized skills to determine the school travel neighborhood environment, and the collection of these data can be feasible (77.8 min on average per school) for researchers and practitioners. There is a need for reliable tools to use for local implementation of SRTS programs, highlighted by the fact that evaluation is one of the least-implemented components of the SRTS Six E’s (engagement, equity, engineering, encouragement, education, and evaluation), and the MAPS-SRTS can be used to both assess the need for specific interventions and document infrastructure changes around schools that are part of SRTS programs [58].

## Conclusions

The MAPS-SRTS audit tool is a reliable instrument for measuring the school travel environment that can be used for a variety of research and evaluation purposes. This tool’s potential importance, once validated, includes being used to evaluate SRTS and tactical urbanistic interventions and to inform policy or advocacy efforts by diagnosing the current condition of school access routes throughout an entire district or area of interest, and identify priority schools for investments in improvements. Additionally, this tool can be used to test the moderating effect of micro-scale factors near schools, on inter-personal/community-based interventions to motivate more children and families to actively commute to school. Finally, this tool can be used to compare and contrast the level of contribution of specific micro-scale factors on active commuting to school behaviors. While there is still further research needed to validate the tool with child ACS behavior and improve the quality of assessment for several constructs, this tool has the potential for use by researchers and practitioners to document and identify areas for intervention around schools to improve safety and active travel for children.

### Electronic supplementary material

Below is the link to the electronic supplementary material.

Supplementary Material 1

## Data Availability

The datasets analyzed during the current study are available upon request to the corresponding author (LAG) and the scoring code and data dictionary for the MAPS-SRTS audit tool is available for those who want to use the tool via https://github.com/LA-Ganzar/maps-srts.

## Abbreviations

ACS: Active commuting to school
GIS: Geographic information systems
ICC: Intraclass correlation coefficients
MAPS: Micro-scale Audit for Pedestrian Streetscapes
MAPS-SRTS: Micro-scale Audit for Pedestrian Streetscapes for Safe Routes to School
SRTS: Safe Routes to School
STREETS: Safe Travel Environment Evaluation in Texas Schools study
