# Supplementary material for: Development, scoring, and reliability for the Microscale Audit of Pedestrian Streetscapes for Safe Routes to School (MAPS-SRTS) instrument
Source: BMC Public Health. 2024 Mar 6;24:722. doi: 10.1186/s12889-024-18202-9 (PMC10916041; doi:10.1186/s12889-024-18202-9)
Supplement: Supplementary file 1 — Supplementary Material 1 [file 12889_2024_18202_MOESM1_ESM.docx]

Microscale Audit of Pedestrian Streetscapes (MAPS), SRTS


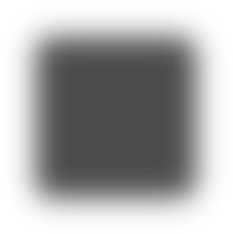

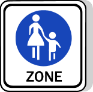


STREETS Training Manual & Picture Guide

**Original Tool and protocol developed by: James Sallis, Lawrence Frank, Brian Saelens, Kelli Cain, Terry Conway, Jim Chapman, Carrie Geremia, Abby King**

**San Diego State University Urban Design 4 Health Children’s Hospital Seattle**

**Stanford University Medical Center**

**Adapted tool and protocol developed by:**

**Deb Salvo, Casey Durand, Leigh Ann Ganzar & Sarah Bentley**

**University of Texas Health Science Center at Houston,**

**Austin Regional Campus**

Table of Contents

[Training Plan 3](#_Toc10730779)

[In the Field 3](#_Toc10730780)

[Supplies: 3](#_Toc10730781)

[Personal Safety 3](#_Toc10730782)

[Expectations 4](#_Toc10730783)

[Tablets 4](#_Toc10730784)

[iPad Survey Administration & Upload Protocol: 4](#_Toc10730785)

[Maps 4](#_Toc10730786)

[Creating the maps for the field 4](#_Toc10730787)

[Defining School Neighborhood Environment Route 6](#_Toc10730788)

[Survey protocol and picture guide 8](#_Toc10730789)

[MAPS SRTS Survey 8](#_Toc10730790)

[School Access Segment 8](#_Toc10730791)

[Crossing 14](#_Toc10730792)

[Segment 20](#_Toc10730793)

## Training Plan

All UTHealth research staff and data collectors should participate in classroom and field training.

Classroom training will include the following:

- Definition of the School Neighborhood Environment Route
- How to create and use maps
- Review of the MAPS training manual and picture guide
- What to expect in the field

Field training will include:

- Hands-on training for each route type (segments, crossings, school access segments)
- Practice using instrument (iPad mini), map and protocol

## In the Field

### Supplies:

- - - Protocol/manual binder
    - A copy of map with assigned street segments
    - Pen
    - iPad
    - Comfortable clothes & shoes
    - Water bottle
    - Cell phone
    - Safety vest (optional)
    - Sun protection, bug spray (optional)
    - Animal/dog deterrent spray (optional)

### Personal Safety

- - - Raters should check weather conditions prior to beginning the audit and prepare accordingly.
    - Raters should conduct audits during daylight hours.
    - If raters feel threatened in any way, they should leave the area immediately and/or call police.
    - COVID-19 prevention: Raters should wear UT ID's (to show we're essential employees), wear face coverings, have only 1 person touch the iPad, and stand 6 feet apart.

### Expectations

- - - Raters will work with their shift partner. One person primarily entering data on a tablet and the other managing the map and protocol. The partners come to agreement as they go through the audit.
    - Raters should take notes on paper (or back of map), especially if there are any edits that will need to be made when returning to the office. These notes should also be entered in the Notes section in Qualtrics.
    - Raters should turn in all tools and maps after each shift in the field whether they have been completed or not.

## Maps

Note: Prior to creating the maps, contact the school coordinator to ask where the main entrance is located. Let them know when we will be in their area. Project director or measurement coordinator will create the maps ahead of time.

### Creating the maps for the field

- We will use the first nearest neighbor method, in which you go two street segments away from the school entrance, or one additional segment past each crossing.
- Segments, crossings, and SAS will be marked with different colored lines.
- Label the school access segment as SAS, each crossing as C1, C2, C3, etc; and each street segment as S1, S2, S3, etc.
  - Numbering for crossings will start with any crossing(s) directly across from the school entrance (if one exists). If there are no crossings in the middle of the school segment, start with crossing to the right (if facing the school) and move clockwise through the crossings. After completing the crossings, move on to the segments around the school in clockwise direction.
  - In the case that a segment on one side of the road ends at T intersection, but the other side continues further, both segments should end at the T intersection.
- Other items your map must include are the identifier code for the school and the names of the staff members doing the audit.
- Special note about driveways/SAS: Assess whether the driveway looks like a private road (longer, gets you from point A to point B, serves the purpose of traveling through it to reach a destination, example Overton Elementary), vs. an actual driveway (pull through, final paved portion not really intended for travel, but rather to park, drop off/pick up people, etc.). Schools should be assessed on a case-by-case basis with input from
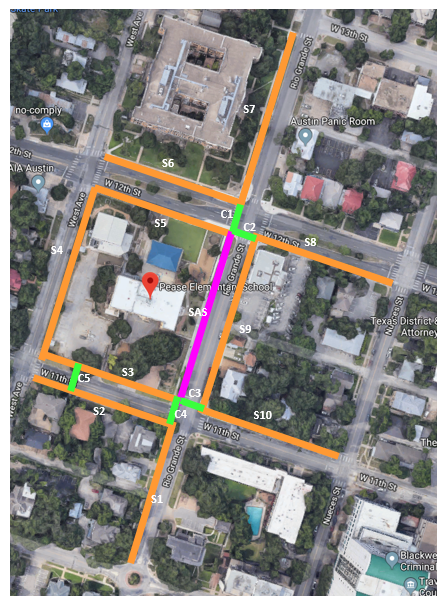
investigators.
- **Sample map:**

## Defining School Neighborhood Environment Route

A **School Neighborhood Environment Route** begins on the street the school is located on or the nearest street available. The school entrance is always the point of reference.

#### Street Segments

A **segment** is a section of street or road between two crossings (one block). We will cover two street segments from the school entrance, known as the first nearest neighbor method.

#### School Access Segments

A School Access Segment is the section of street directly in front of the main school entrance between two intersections; intersections are determined by using both sides of the road (ie, center of the road); for example, if there are no intersections on the SAS (school) side, but there are T intersections on the other side, these would count as intersections. The SAS should cover the entire front of the school building (at least including any places children can access the entrance). There may be more than one SAS if there is an intersection across from the entrance of the school. A crossing should be added at each of these intersections, even if there is no official crosswalk. This would constitute the end of one SAS and beginning of another. Driveways on the SAS (school side) do NOT divide the SAS into two.

#### Crossings

A **crossing** occurs when the rater must go through an intersection, whether a pedestrian crossing exists or not. Crossings are located between two segments. A crossing should be added any place two roads intersect. A driveway along a segment cannot be considered as a crossing, however a driveway in the SAS will be audited as a crossing. Many streets may not have any crossings (e.g., long suburban road).

***Unanticipated crossings guidance:*** During the course of the audits, you may encounter a need to make unanticipated crossings along a segment. An unanticipated crossing is one where you must stop and cross to the other side of the street due to a permanent or semi-permanent obstruction* in order to continue moving forward.

In general, you should follow the guidelines in the protocol: If you encounter an obstruction, stop auditing the segment you are on. Cross over to the other side of the street, complete a crossing audit for that ad-hoc crossing, and start a new segment. Continue walking on the new segment in the same direction as before, and **only record features present on the new side of the street from the point of the crossing on forward (as you would with any segment audit)**.

**However,** if your unanticipated crossing takes you onto a segment that **you were assigned to audit anyway**, simply end the segment with the obstruction, complete the audit for the unanticipated crossing, **and then do the full audit of the other side of the street you were going to do anyway (meaning from intersection to intersection, as usual).**

When you must perform an audit of an unanticipated crossing, instead of intersections, you should record the address of the house or building you started the crossing with and that you ended the crossing with. If there is no address because no buildings face onto the segment where you crossed, then put a note to indicate about how far down the segment you were (10%, 20%, 30%, etc.).

**An obstruction is one that impedes your ability to continue moving forward. If you can step around it and remain on the sidewalk, then it is not an obstruction. If you must step into the street or on to private property to get around it, then it is an obstruction.* ***The obstruction must be permanent or semi-permanent.*** *A car that happens to be blocking a driveway at that moment is not permanent or semi-permanent. Nor is temporary construction (e.g. sidewalk closed to repair a power line), a fallen tree branch, etc. Semi-permanent would be something like building construction that has shut down the sidewalk on that side of the street for the foreseeable future. Essentially, any obstruction that could or will be moved within a few days should not be considered permanent or semi-permanent.*

## Survey protocol and picture guide

### MAPS SRTS Survey

There are 3 sections to the Safe Routes to School portion of the tool: School Access Segment (SAS), Crossing, and Segment.

Raters will always start by walking around the school block to confirm the map is correct and mark any additional crossings if encountered. Then, begin the rating at the school access segment. After completing the school access segment, start with any crossing(s) across from the school entrance (if one exists). If there are no crossings in the middle of the school segment, start with crossing to the right (if facing the school) and move clockwise through the crossings. After completing the crossings, move on to the segments around the school in clockwise direction.

## School Access Segment

*Refer to segment section for photos and explanations.*

##### For this section, count your side of the street.

Street:

_________________________________________

Side: (choose from)

North

South

East

West

Starting Cross-street:

_________________________________________

Ending Cross-street:

_________________________________________

1. Are there public transit stops present?
   - Yes
   - No
2. How many low streetlights (for people walking) are installed?
   - None
   - Some
   - Ample
3. How many high streetlights (for cars) are installed?
   - None
   - Some
   - Ample
4. What percentage of the length of the sidewalk/walkway is adjacent to a parking lot?
   - 0%
   - 1-49%
   - 50-99%
   - 100%
5. Are there signs of neglect, such as graffiti or poorly maintained buildings or abandoned buildings?
   - None
   - Some
   - Ample
6. Is there a designated bike lane or path? *(Check one)*
   - No
   - Sharrow sign in street
   - Painted line
   - Green or other painted color lane
   - Physical barrier of parked cars (between roadway and marked bike lane)
   - Other physical barrier (on street or off street)
7. Is a continuous sidewalk present?
   - Yes, paved sidewalk is continuous
   - No, paved sidewalk is not continuous
   - No paved sidewalk, but informal waling path or wide (≥5’) road shoulder
   - No, no sidewalk or informal walking path
8. What is the width of the majority of the sidewalk?
   - < 3ft
   - 3-5 ft
   - > 5 ft
   - No sidewalk
9. Are there poorly maintained sections of the sidewalk that constitute major trip hazards? (e.g. heaves, misalignment, cracks, overgrowth, incomplete sidewalk) – (Check one)
   - None
   - One A few
   - A lot
   - No sidewalk
10. Is a buffer present (sidewalk separated from roadway by a parking lane, regularly planted trees, landscaping or other barrier)?
    - No
    - Yes
    - No sidewalk
11. What percentage of the length of the sidewalk/walkway is covered by tree canopy?
    - 0-25%
    - 26-75%
    - 76-100%
    - No sidewalk/walkway
12. What percentage of the length of the sidewalk/walkway is covered by awnings or other overhead coverage?
    - 0-25%
    - 26-75%
    - 76-100%
    - No sidewalk/walkway

##### School zone signage characteristics


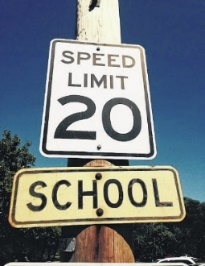


At All Times


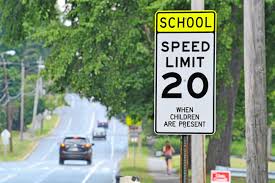


When children are present

1. Is there a posted speed limit on this segment?
   - ≤15 MPH
   - 16-25 MPH
   - 26-35 MPH
   - 36-49 MPH
   - 50+ MPH
   - None


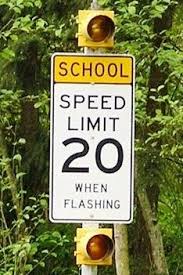

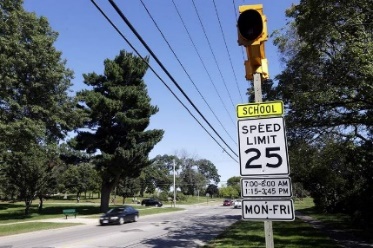


Certain time-periods only

1. What are the extent of any speed restrictions in the special speed zone? *(Check one)*
   - None noted
   - Restrictions applicable at all times
   - Restrictions applicable for certain time periods only
   - Restrictions applicable when children are present
     1. Is there any signage with flashing lights?


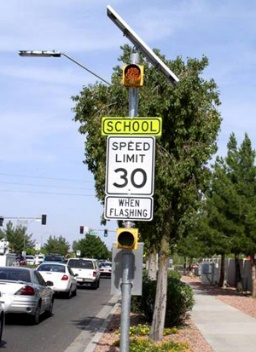


Signage with flashing lights


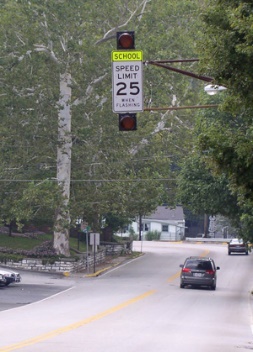


Signage hanging over street

- - Yes
  - No
    1. Is there any signage hanging over the street/roadway?
  - Yes
  - No

##### Traffic Calming features

- - 1. Are there traffic-calming circles present?
  - Yes
  - No
    1. Are there speed tables present?
  - Yes
  - No
    1. Are there speed humps present?
  - Yes
  - No
    1. Are there any narrowing streets?
  - Yes
  - No

##### Roadway signage

- - 1. Are there instructional signs for people walking (identifies to the pedestrian where to walk or cross the street?
  - Yes
  - No
    1. Are there instructional signs for people biking (identifies to the cyclists where to bike or cross the street)?
  - Yes
  - No
    1. Is there crosswalk signage or other pedestrian signage (for drivers; alerts drivers where pedestrians crossing)?
  - Yes
  - No
    1. Is there cycling signage (for drivers; alert drivers where cyclists may be biking)?
  - Yes
  - No
    1. Is there signage warning the drivers of presence of children?
  - Yes
  - No
    1. Is there school zone signage?
  - Yes
  - No

*Consider all signage, including parking signs*

1. Is roadway signage well maintained? (Check all that apply)
   - Overgrown bushes/tree branches obscure signage
   - Poorly maintained (unclean) street/stop/speed zone or other signs
   - Signs are missing or damaged
   - None (signage is visible and readable)
   - N/A (no signs)
2. Land use type (Check all that apply)
   - Residential
   - Non-residential
   - Public park
   - School

*Daycare or Montessori school is non-residential, not school*

##### Street Amenities

- - 1. Are there trash bins (public)?

*Only count amenities for public use (not on private property); and if on government property, must be within 5 feet of the edge of the street or sidewalk to count as "public use".*

***Do not*** *double-rate bus stop benches; they will be counted under the previous section on public transit stops.*

- - Yes
  - No
    1. Are there benches or other places to sit?
  - Yes
  - No
    1. Are there bicycle racks?
  - Yes
  - No

1. How many driveways are there? Count only segment side of the street.
   - None
   - 1-2
   - 3-5
   - 6+
2. Is there a school driveway or parking lot entrance or exit that crosses the sidewalk/walkway along the segment? (For each driveway present, complete a “Crossing” assessment)
   - None

*For A22, only count school driveways. If there are private drives along the SAS, we do NOT need to do a crossing.*

- - 1
  - 2+

Notes:

_________________________________________

## Crossing

For this section, you will only rate the crossing *nearest* to the school access point.

##### Crossing Location

- - 1. Is the crossing located at an entrance/exit driveway on school property?
  - Yes 🡪 fill out below, then you will be directed to question C2
  - No 🡪 go to question C1b

The crossing is on the block of ___________ (Example: 5^th^ St., Rose Blvd., etc.)

_________________________________________

between

_________________________________________

and

_________________________________________.

- - 1. Is the crossing located at a crossing adjacent to school property (crossing to the school property)?
  - Yes 🡪 go to question C1bi
  - No 🡪 go to question C1c
    - 1. Is the crossing located at an intersection?
  - Yes 🡪 fill out below and go to question C2a
  - No 🡪 go to question C1bii

The crossing is on the block of ___________

_________________________________________

and

_________________________________________.

*“Put yourself in the body of a kid walking to school.” – Dr. Casey Durand*

Crossing from *(pick one)*

North

South

East

West

to *(pick one)*

North

South

East

West

- - - 1. Is the crossing located in the middle of the road?
  - Yes 🡪 fill out below and go to question C2a
  - No 🡪 go to question C1c

The crossing is on the block of ___________ (Example: 5^th^ St., Rose Blvd., etc.)

_________________________________________

between

_________________________________________

and

_________________________________________.

- - 1. Is the crossing located at another location not included in the two options above?
  - Yes 🡪 go to question C2a
  - No 🡪 go to question C2a


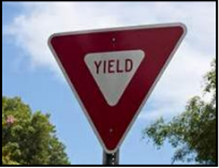


**Yield signs:** indicate right-of-way


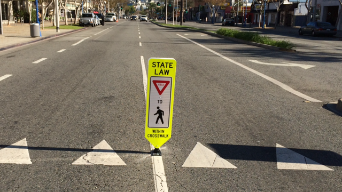


##### Intersection Control


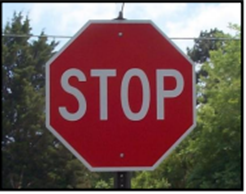


**Stop signs:** ensure that vehicles will stop for a certain period of time to allow other vehicles, pedestrians, and non-motorized vehicles to cross the intersection

- - 1. Are there any yield signs?
  - Yes
  - No
    1. Are there any stop signs?
  - Yes
  - No
    1. Are there any traffic signals?

**Traffic signal:** a visual signal to control the flow of traffic at intersections

- - Yes
  - No


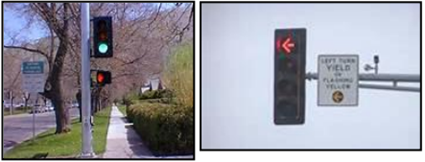


- - 1. Are there any traffic circles?
  - Yes
  - No


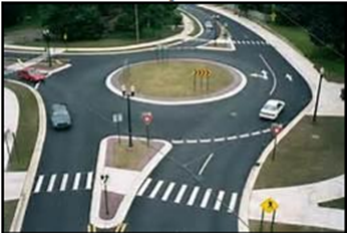


**Traffic circle:** requires all traffic to travel in one direction around a central island

#####
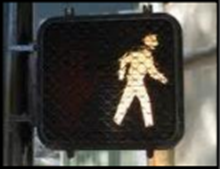

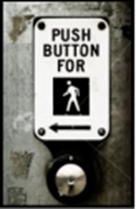
Pedestrian and Bicycle Signals

**Push button:** Actual button for pedestrians to push to indicate they are waiting to cross

- - 1. Are there any pedestrian walk signals?
  - Yes
  - No

**Pedestrian walk signals:** some indication for pedestrians to know when to walk

- - 1. Are there any push buttons?
  -
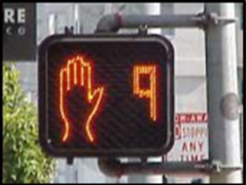
Yes
  - No
    1.
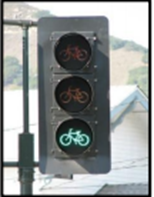
Are there any bicycle signals?
  - Yes
  - No
    1. Are there any countdown signals?

**Countdown signal:** both pedestrian triggered and automatic signaling systems are programmed to indicate safe crossing for specified periods of time

- - Yes

**Bicycle signal:** signal specifically for bicycles using the crossing

- - No

##### Curb Characteristics

***Pre-curb is always the one closest to the school entrance.***

- - 1. Pre-crossing curb *(Check one)*
  - Ramp lines up with crossing
  - Ramp does not line up with crossing
  - No ramp or no curb (including when a sidewalk is no present)
    1. Post-crossing curb *(Check one)*
  - Ramp lines up with crossing
  - Ramp does not line up with crossing
  - No ramp or no curb (including when a sidewalk is no present)

#####
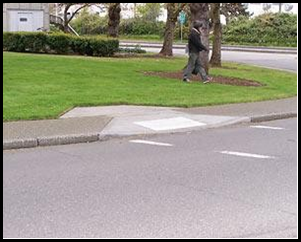

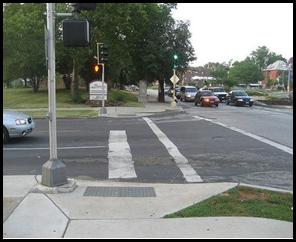
 Crosswalk Treatment

No ramp

Ramp doesn’t line up with crossing

Ramp lines up with crossing


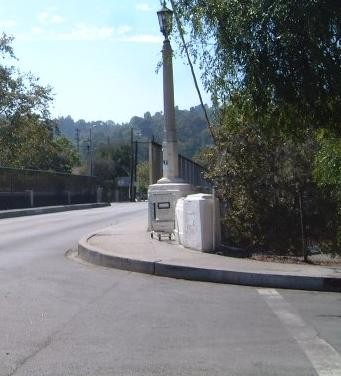

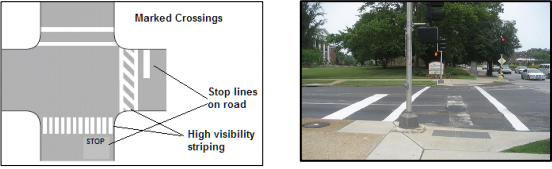

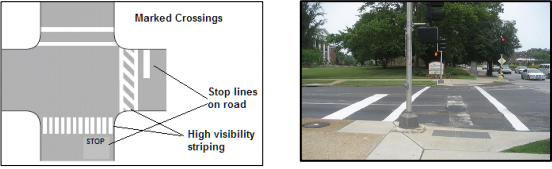


- - 1. Is there a marked crosswalk?
  - Yes
  - No
    1. Is there high-visibility striping?

**Marked Crosswalk:** A crosswalk is a designated point on a road at which some means are employed to assist pedestrians wishing to cross. They are designed to keep pedestrians together where they can be seen by motorists, and where they can cross most safely with the flow of vehicular traffic. Pedestrian crossings are often at intersections, but may also be at other points on busy roads that would otherwise be perilous to attempt to cross.

- - Yes
  - No
    1. Is there a raised crosswalk?
  - Yes


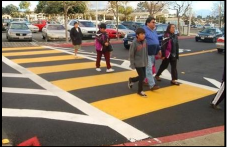


**High-Visibility Striping:** Usually indicated by ladder or diagonal striping or unique lighting, striping for the crosswalk that is more visible to drivers than simple parallel lines. (Example: 2 yellow lines or zebra stripes would count here)

- - No
    1. Is there different material than road?
  - Yes
  - No


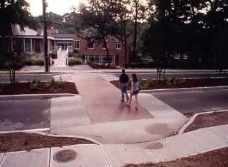


**Different Material than Road:** Crosswalks characterized by variations in the material along the crosswalk that distinguishes it from the street portion dedicated to vehicular traffic.


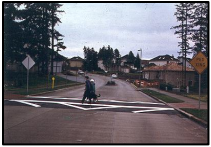


**Raised Crosswalk**: Indicate the crossing path by a surface slightly elevated from the street pavement

*Different material: Yes - concrete crosswalk if street is asphalt*

#####
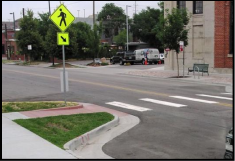

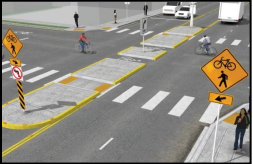
Features

- - 1. Are there protected refuge islands?
  - Yes
  - No

Protecte refuge islands

Curb extension

- - 1. Is there a curb extension present?
  -
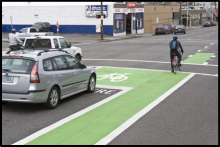
Yes
  - No

Bike box

- - 1. Is there a bike box present?
  - Yes
  - No

***Pedestrian refuge*** *could be a median that’s clearly for someone to stand in (i.e., has concrete pad on end)*

***Curb extensions*** *may run down block face; auditors should look not just at the immediate corner area, but the full block face to determine if curb extensions are present.*

##### Travel Lanes Walked Through

1. How many travel lanes does a person walking have to cross?


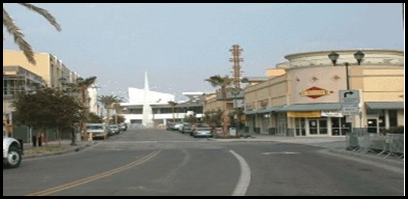
1


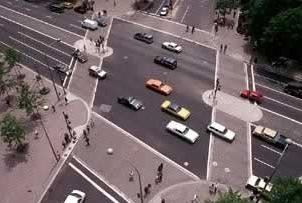


2

3

4

5

6

7+

8 Lanes

4 Lanes including a turn & parking lane


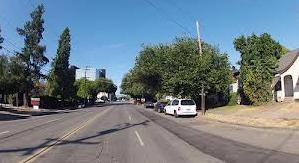
**Number of traffic lanes:** Count travel lanes and vehicle parking lanes. Examples of width: This question is trying to get at the size of the street, so if there is parking allowed on each side of the street and 2 cars can pass each other at the same time, this would be counted as 4. If 2 cars cannot pass each other with parking available on both sides of the street, it will be counted as 3 lanes.

6 Lanes residential

Dedicated turn lane | Dual Turn lane
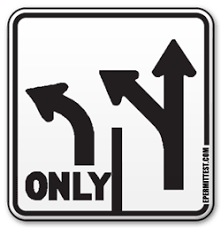


##### Turn Lanes Walked Through

1. How many turn lanes does a person waling have to cross? ***Do not include travel, dual or parking lanes.***
   - 0
   - 1
   - 2
   - 3+

##### Miscellaneous Problems

1.
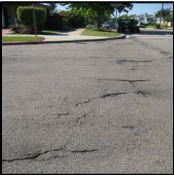

   - 1. Is the crossing surface in poor condition?
   - Yes
   - No
     1. Are there any faded or worn crosswalk markings?
   - Yes
   - No
     1. Are there **no** crosswalk markings?

**Poor Condition of Crossing Surface:** Pedestrian would need to look down at the ground to feel confident that they could cross without encountering a trip hazard.

- - Yes
  - No


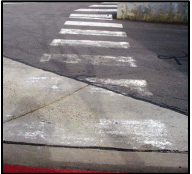


**Faded/Worn Crosswalk:** Majority of crosswalk within direct route of traffic is **not** clearly visible.

Notes:

_________________________________________

## Segment

Street:

_________________________________________

Side: (choose from)

North

South

East

West

Starting Cross-street:

_________________________________________

Ending Cross-street:

_________________________________________

1. Are there public transit stops present?
   - No
   - Yes

Low Street Light


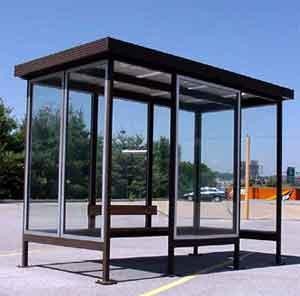


Transit stop

Bus stop


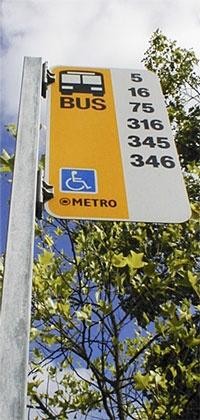


1. How many low street lights (for people walking) are installed?
   -
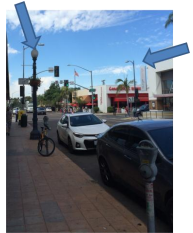
None
   - Some

High Street Light

- - Ample

1. How many high street lights (for cars) are installed?
   - None
   - Some
   - Ample
2. What percentage of the length of the sidewalk/walkway is adjacent to a parking lot?

*Parking lot must have an entrance and/or exit onto the segment. A small grass (or other) buffer is okay. Lot may be only a few spaces.*

- -
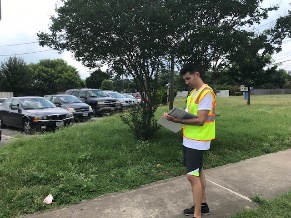
0%
  - 1-49%
  - 50-99%
  - 100%

1. Are there signs of neglect, such as graffiti or poorly maintained buildings or abandoned buildings?

*You should be able to justify any neglect with specific examples. Buildings do not need to be brand new to get a rating of none. They just need to be well kept and maintained.*

- - None
  - A little (present)
  - Some (very noticeable)
  - A lot (overwhelming)


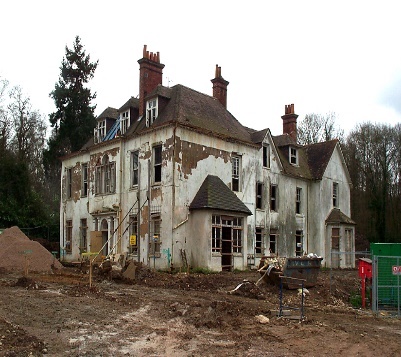


A lot (overwhelming)


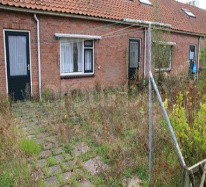


Some (Very noticeable)


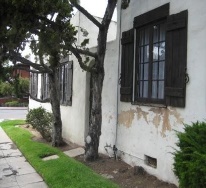


A little (present)


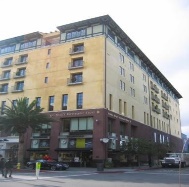


None

1. Is there a designated bike lane or path? *Check one*
   - No
   - Sharrow sign in street
   - Painted line
   - Green or other painted color lane
   - Physical barrier of parked cars (between roadway and marked bike lane)
   - Other physical barrier (on street or off street)


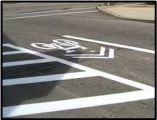

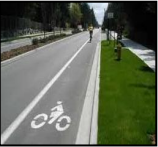

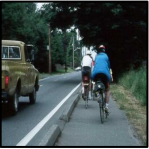

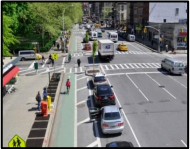


Sharrow sign in street

Painted line

Physical barrier

Physical barrier of cars

1. Is a continuous sidewalk present?
   - Yes. Paved sidewalk is continuous


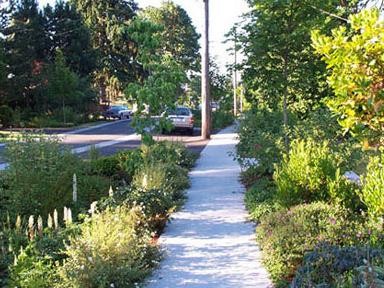


Continuous

- - No. Paved sidewalk is not continuous
  - No paved sidewalk, but informal walking path or wide (≥5’) road shoulder
  - No. No sidewalk or informal walking path

**Non-continuous sidewalk:** A sidewalk that stops mid-segment or interrupted (i.e. by an alley).

*- If the sidewalk (at any point) is so narrow that it is not passable with a stroller, then it is non-continuous.*

*- Steps (in good condition) would NOT be said to interrupt a sidewalk. Our primary interest is pedestrians, not cyclists (on the sidewalk).*


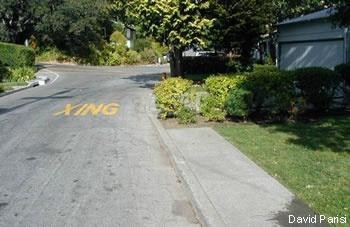


Non-continuous

1. What is the width of the majority of the sidewalk?
   - < 3 ft.
   - 3-5 ft.

Reference Note: a size 8 U.S. shoe size is equivalent to 9.5 inches!

- - > 5 ft.
  - No sidewalk


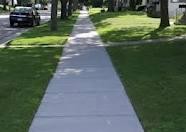

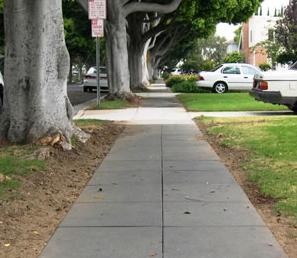


**3-5 ft**

**< 3 ft**


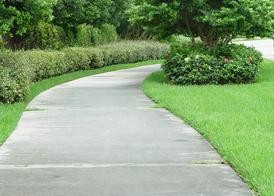


**> 5 ft**

1. Are there poorly maintained sections of the sidewalk that constitute major trip hazards? *(e.g. heaves, misalignment, cracks, overgrowth, incomplete sidewalk)
   Check one*

*If you are having trouble deciding, imagine you are pushing a stroller (with regular wheels). Would the issues cause a problem?*

***Cobbled stone*** *(in good condition) would NOT be a problem.*

*Condition of driveway or alley are NOT part of sidewalk.*

- - None
  - One
  - A few
  - A lot
  - No sidewalk

Trip Hazard: An increased likelihood of tripping due to a raising or lowing in the walkway. A hazard could be due to plants, tree roots, or general erosion. Major trip hazards would require walkers to look down in order to avoid tripping.


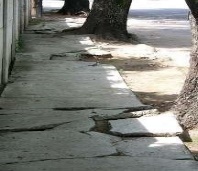


A lot


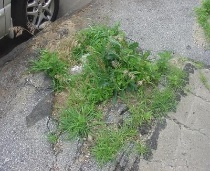


One


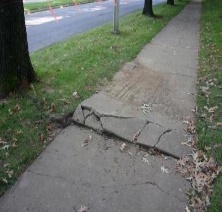

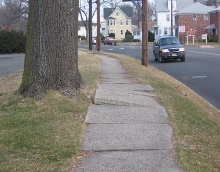


A few

**Heave**: Uneven or raised portion of the sidewalk that could be a trip hazard, usually caused by tree roots or soil expansion after a period of frost.

1. Is a buffer present (sidewalk separated from roadway by a parking lane, regularly planted trees, landscaping, or other barrier)?
   - No
   - Yes
   - No sidewalk


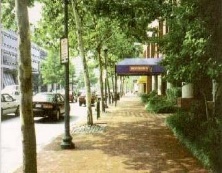


**Tree buffer**


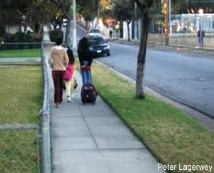


**Grass buffer**


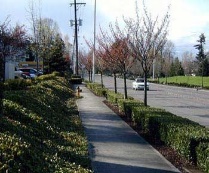


**Shrub buffer**


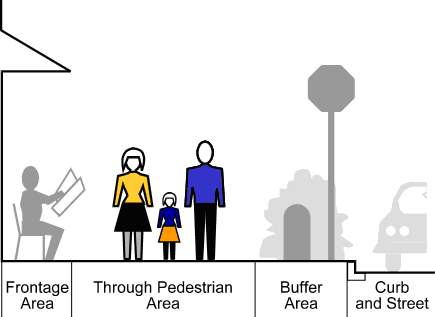

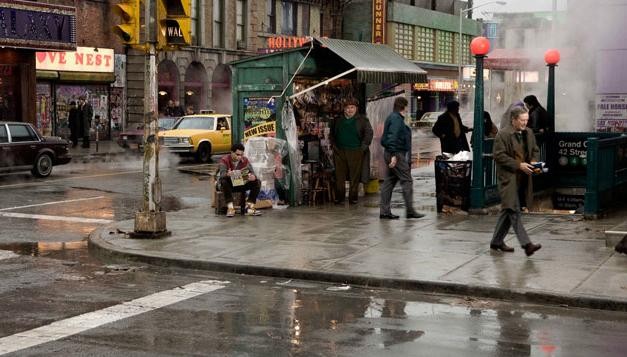


**No buffer**


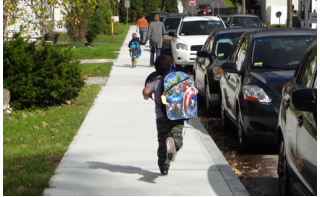


**Car buffer**

**Buffer:** Separates vehicular and pedestrian zones parallel to the edge of paved roads. They often occupy space between traffic lanes and walking paths that is not intended for either vehicle traffic or walkers. Any buffer on a segment, no matter how long, will be counted.

- - - Tree plantings, telephone poles or parking meters should not be considered as a buffer if there is, on average, more than 20 feet between them along the street segment.
    - A bike lane does not count as a buffer.
    - Brick or other flat material alone next to a sidewalk would not be counted as a buffer because it is not inhibiting cars from coming onto the sidewalk.

*Count cars as a buffer if:*

*• There are parking lines or signage, whether there are cars there or not at that moment,*

*• OR, it’s a place where cars would generally park, whether there are cars there or not at that moment,*

*• OR, there are cars parked there at that moment, whether there are signs/lines or not.*

*• Do NOT count if a car is there at that moment but parking is specifically prohibited by signage (even during specific times – like drop-off / pickup times)*


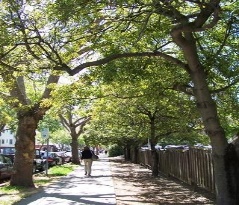

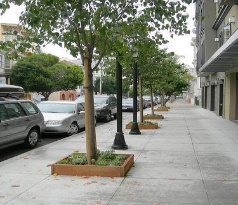


26-75% Coverage

76-100% Coverage

1. What percentage of the length of the sidewalk/walkway is covered by tree canopy?
   - 0-25%
   - 26-75%
   - 76-100%
   - No sidewalk/walkway

**Tree Coverage:** Tree Coverage is the percent of walkway covered by trees, awnings, or other structures providing shade to the walkway. It need not cover the entire width of the sidewalk. Depending on the time of the year, trees may lose their leaves, so make sure to visualize the trees with their full foliage.

1. What percentage of the length of the sidewalk/walkway is covered by awnings or other overhead coverage?
   - 0-25%

*Sidewalk here refers to the pathway; it does not have to be paved.*

- - 26-75%
  - 76-100%
  - No sidewalk/walkway
    1. Is there a posted speed limit on this segment (non-school zone, normal street, etc.)?


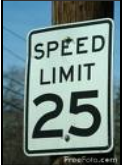


Non-school zone

*If multiple, select the highest*

- - ≤15
  - 16-25
  - 26-35
  - 36-49
  - 50+
  - None
    1. Is there a posted speed limit on this segment (special zone-school, etc.)?


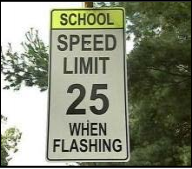


Special zone; “end school zone” does not count

*If multiple, select the highest*

- - ≤15
  - 16-25
  - 26-35
  - 36-49
  - 50+

##### Traffic Calming Features

- Count each traffic calming indication separately. Example: a speed bump accompanied by a sign indicating the bump would count as 2
- Dip in the road accompanied by a sign to alert drivers should be counted
- Dip in the road without a sign should not be counted
- A guardrail does NOT count as traffic calming
  - 1. Are there any traffic calming circles?

**Traffic Calming:** Infrastructure with the purpose of reducing vehicle speeds and improving safety for drivers and pedestrians (e.g., traffic calming signs, traffic circles, speed tables, speed humps, curb extensions, traffic lights). Designed measures compel drivers to slow down, or act to exclude or divert traffic altogether.

- - Yes
  - No
    1. Are there any speed tables?
  - Yes
  - No
    1. Are there any speed humps?
  - Yes
  - No
    1. Are there any narrowing streets?
  - Yes
  - No


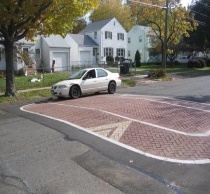

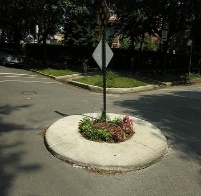

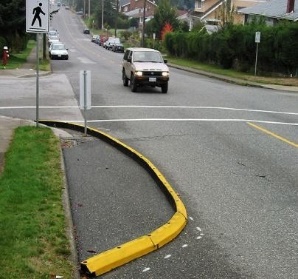

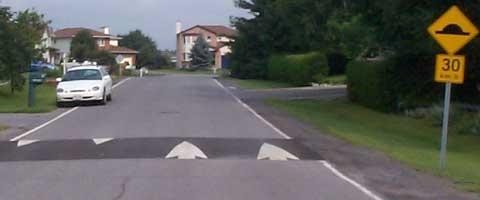


Speed hump

Speed table

Traffic circle

Street narrowing

##### Roadway Signage


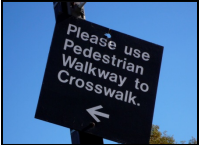

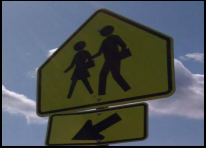


**a. Instructional Signs for People Walking:**
Identify to the pedestrian where to walk or cross the street. These are sometimes positioned closer to the crosswalk and may have an arrow pointing down.

- - 1. Are there instructional signs for people walking (identifies to the pedestrian where to walk or cross the street)?
  - Yes
    - - - No
    1. Are there instructional signs for people biking (identifies to the cyclist where to bike or cross the street)?


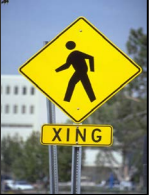

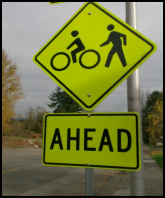


**b. Crosswalk Signage or Other Pedestrian Signage (For Drivers):** Alerts drivers where pedestrians may be crossing.

- - - - - Yes
        - No
    1. Is there crosswalk signage or other pedestrian signage (for drivers; alerts drivers where pedestrians may be crossing)?
       - - Yes
         - No


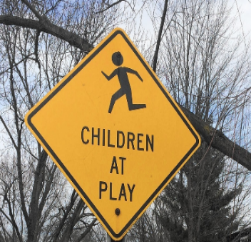


**c. Signage Warning Drivers of Presence of Children**

- - 1.
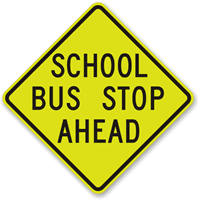
Is there cycling signage (for drivers; alerts divers where cyclists may be biking)?
       - - Yes
         - No
    2. Is there signage warning drivers of the presence of children?

**f. School Zone Signage**End School Zone signage does not count.


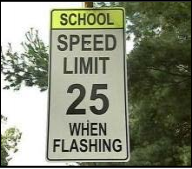


- - - - - Yes
        - No
    1. Is there school zone signage?
       - - Yes
         - No

1. Is the roadway signage well maintained? (*Check all that apply)*
   - Overgrown bushes/tree branches obscure signage
   - Poorly maintained (unclean) street/stop/speed zone or other signs
   - Signs are missing or damaged
   - Yes (signage is visible and readable)
   - N/A (no signs)
2. Land Use Type: (*Check all that apply)*
   - Residential
   - Non-residential
   - Public park
   - School


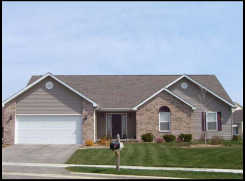


**Single-family home**


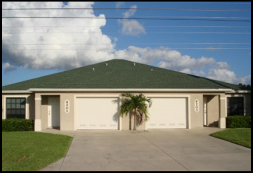


**Multi-unit home**


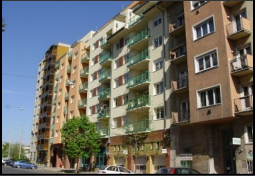


**Apartments**

**Residential** (Single-family homes, multi-unit homes, apartments, etc.)


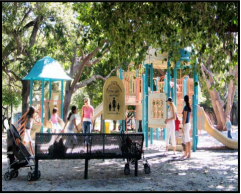

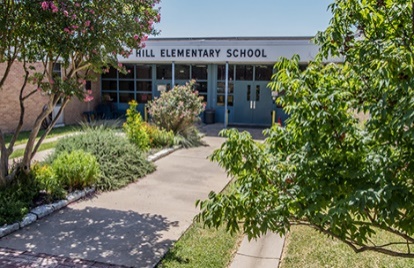


**Public Park** (playground, dog park, etc.)

**School** (elementary school)


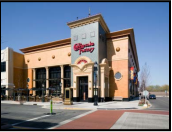


Sit down-down restaurant


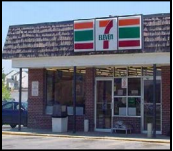


Convenience store


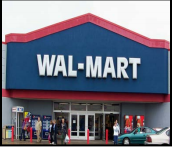


Big box retail


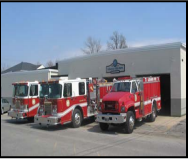


Government (Fire station, etc.)

**Non-residential** (food related, retail and service oriented land uses, government, etc.)

*Daycare or Montessori school is non-residential, not school*

##### Presence of Street Amenities


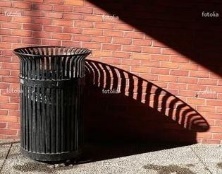


Trash bin: must be for public (or pedestrian) use, not private residences’ trash bins

- - 1. Are there trash bins (public)?
       - - Yes
         - No
    2. Are there benches or other places to sit?
       - - Yes
         - No
    3. Are there bicycle racks?
       - - Yes
         - No

**Benches or other places to sit:** Tables or benches outside of restaurants/cafés do not count as a street amenity (places to sit). These need to be public seating areas.


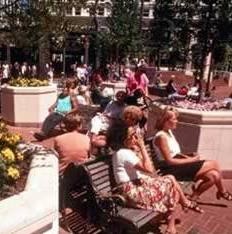

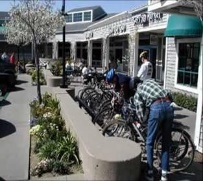


**Bicycle racks**

*Only count amenities for public use (not on private property); and if on government property, must be within 5 feet of the edge of the street or sidewalk to count as "public use".*

***Do not*** *double-rate bus stop benches; they will be counted under the previous section on public transit stops.*

1. How many driveways are there? *Count only segment side of the street*.
   - None
   - 1-2

***Driveways:*** *Count anything that interrupts the sidewalk, like an alley or anything else where cars may frequently go in/out.*

- - 3-5
  - 6+
